# Ruxolitinib Reduces Oxidative Stress in Patients With Primary Myelofibrosis: A Multicenter Study

**DOI:** 10.7759/cureus.20929

**Published:** 2022-01-04

**Authors:** Mahmut B Koyuncu, Mustafa Ilgan, Hakan Basir, Anil Tombak, Mehmet Ali Ucar, Tolga Koseci, Aydan Akdeniz, Eyup N Tiftik, Özcan Erel

**Affiliations:** 1 Hematology, Adana City Research and Training Hospital, Adana, TUR; 2 Internal Medicine, Mardin State Hospital, Mardin, TUR; 3 Internal Medicine, Mersin University, Faculty of Medicine, Mersin, TUR; 4 Hematology, Mersin University, Faculty of Medicine, Mersin, TUR; 5 Hematology, Cukurova University, Faculty of Medicine, Adana, TUR; 6 Medical Oncology, Adana City Research and Training Hospital, Adana, TUR; 7 Biochemistry, Yıldırım Beyazit University, Ankara, TUR

**Keywords:** disulfide, oxidative stress, ruxolitinib, thiol compounds, primary myelofibrosis

## Abstract

Introduction

Primary myelofibrosis (PM) has a lower overall survival rate than other myeloproliferative neoplasms, and leukemic transformation is the most common cause of death. Increased oxidative stress has an important role in leukemic transformation in these patients. In this study, we aimed to find an answer to the question, "Could Ruxolitinib, which has been widely used in patients with myelofibrosis in recent years, have a role in reducing oxidative stress in these patients?".

Methods

A total of 106 patients with PM and 111 healthy volunteers were included in this study. We collected the serum samples of healthy volunteers and patients with myelofibrosis at the time of diagnosis and one month after the initiation of Ruxolitinib treatment. Ischemia modified albumin (IMA), native thiol, total thiol, and disulfide levels were studied. The disulfide/native thiol, disulfide/total thiol, and native thiol/total thiol ratios were calculated.

Results

IMA, native thiol, total thiol, disulfide levels, disulfide/native thiol, and disulfide/total thiol ratios at the time of diagnosis were significantly different in patients with myelofibrosis compared to the control group (p=0.001). Ruxolitinib significantly reduced oxidative stress when the measurements in the first month after Ruxolitinib were compared with those at the time of diagnosis (p=0.001). In patients with ASXL1 mutation, intermediate-2 risk, and high-risk according to the Dipps-plus score, the decrease in oxidative stress in the first month of treatment was more significant than at the time of diagnosis.

Conclusion

Ruxolitinib may be an effective treatment for reducing oxidative stress in patients with PM. The reduction in oxidative stress parameters with treatment in patients with ASXL1 mutation, intermediate-2, and high-risk patients was observed to be higher.

## Introduction

Primary myelofibrosis (PM) is a rare but highly mortal myeloproliferative neoplasm characterized by clonal proliferation of myeloid cells [1]. The most common cause of death is the leukemic transformation that develops in approximately 20% of these patients [2]. Unlike de novo acute leukemias, median survival is less than three months [3]. In most patients with PM, either of the Janus kinase 2 (JAK2), CALR, or MPL mutations is present. The 'triple-negative' group represents patients without any of these mutations. The most common epigenetic mutation is the ASXL1 mutation. 'Triple-negative' patients having the ASXL1 mutation demonstrate poor prognosis, and the leukemic transformation is more frequent [4]. A dynamic international prognostic scoring system plus (DIPSS-plus) score is commonly used to determine prognosis and predict leukemic transformation in patients with myelofibrosis. According to this score, patients are divided into four prognostic groups: low risk, intermediate-1 risk, intermediate-2 risk, and high risk. Since the number of high-risk mutations defined in MF disease is increasing, the use of new prognostic scores such as GIPSS (genetically-inspired prognostic scoring system) and MIPSSv2 (mutation-enhanced international prognostic scoring system version 2.0) is becoming increasingly common [1]. Ruxolitinib is an orally administered JAK inhibitor that provides a survival advantage, especially in intermediate-2 risk and high-risk patients; it facilitates the attenuation of constitutional symptoms and reduces the spleen size independent of JAK2 mutation status [5].

It has been shown in various studies that oxidative stress plays a crucial role in both the pathogenesis of PM and the transformation of acute leukemia by causing DNA damage [6]. Administration of Ruxolitinib has been observed to reduce oxidative stress in various rheumatological disorders [7]. In their study, Erel and Neselioglu demonstrated successful results using a new method to measure oxidative stress in many diseases: the thiol and disulfide hemostasis technique [8]. Ischemia modified albumin (IMA), produced when free radicals modify albumin under oxidative stress induced by an ischemia-like environment, can be used as an indicator of increased oxidative stress [9]. In this study, we aimed to review the status of IMA and thiol/disulfide balance in patients with myelofibrosis at the time of diagnosis and demonstrate the changes after Ruxolitinib treatment for the first time in the literature.

## Materials and methods

This is an observational study that included 111 healthy volunteers and 106 patients with primary myelofibrosis over 18 years of age, diagnosed at the Hematology Departments of Mersin University, Adana City Training and Research Hospital, and Cukurova University and treated with Ruxolitinib between January 2020 and July 2021. Ruxolitinib was started in all patients at a standard dose of 20 mg twice a day. Dose adjustments were made according to weekly platelet results. All patients used Ruxolitinib for a month without interruption. Cases with inflammatory diseases (infection, autoimmune disease, solid cancer, etc.), using antioxidant drugs (statins, vitamins, etc.), and prefibrotic cases were excluded from the study. Patients' information was collected by chart review. A PM diagnosis was made according to the 2016 diagnostic criteria of the World Health Organization. Patients' demographic characteristics, their ultrasonographic longitudinal spleen dimensions at the time of diagnosis, routine laboratory results, myelofibrosis-related mutations, and their risk categories determined according to their DIPSS-plus scores were recorded.

This study was carried out in accordance with the principles of the Helsinki Declaration and was approved by the Clinical Research Ethics Committee of Mersin University (date and decision no: 2020/575). Written informed consent of all patients and volunteers was obtained.

Biochemical measurements

The blood samples of the patients were taken at the time of diagnosis and in the first month after starting the Ruxolitinib treatment, and the blood samples of healthy volunteers were taken only once. The IMA measurements were performed by the ELISA method, and the results have been shown in the ng/mL unit. The thiol and disulfide measurements were performed with the new method that Erel and Neslioglu developed [8]. Total thiol, native thiol, and disulfide levels were shown in mmol/L. The native thiol/total thiol, disulfide/total thiol, and disulfide/native thiol ratios were calculated and displayed as percentages (%). 

Statistical method

For statistical analysis, NCSS (Number Cruncher Statistical System; Kaysville, Utah, USA) software was used. Descriptive statistical methods (mean, standard deviation, median, frequency, ratio, minimum, maximum) were used to evaluate the study data. We used Kolmogorov-Smirnov, the Shapiro-Wilk test, and graphical evaluations to test the suitability of quantitative data for normal distribution. For two-group comparisons of normally distributed quantitative data, the Student's t-test was used. Mann Whitney The U test was used to compare two groups of data that did not have a normal distribution.For the comparison of three or more normally distributed groups, the one-way anova test was used; for their paired comparisons, the Bonferroni test was used; for the comparison of three or more groups that do not show normal distribution, Kruskal Wallis test was used; and in pairwise comparisons, Bonferroni-Dunn test was used. We used Fisher's Exact Test in the comparison of qualitative data. For evaluating the relationships between variables, we used Pearson Correlation Analysis for variables that distributed normally, and Spearman's Correlation Analysis for variables that did not distribute normally. Paired Sample t-test was used for intragroup comparisons of parameters that showed normal distribution. The Wilcoxon Signed Ranks Test was used for intragroup comparisons of parameters that did not show normal distribution. P <0.05 is considered statistically significant.

## Results

In this study, we included 111 healthy volunteers and 106 patients with PM. All of the patients were overtly fibrotic. While the mean age of the patients was 62.21±10.09 years, the mean age of the control group was 62.32±11.18 years (p=0.941). Table 1 shows the general characteristics of the patients, the distribution of the risk groups according to the DIPSS-plus scores, and the laboratory findings.

**Table 1:** Distribution of PM group properties DIPSS: Dynamic International Prognostic Scoring System, PM: primary myelofibrosis, SD: standard deviation

		PM (n=106)	
		n	%
Genetic mutation status	Triple negative	6	5.7
	JAK2 (+) ASXL1 (−)	45	42.6
	MPL (+) ASXL1 (−)	10	9.4
	CALR (+) ASXL1 (−)	18	17.0
	ASXL1 (+)	27	25.5
DIPSS-plus category	Low risk	19	17.9
	Intermediate-1 risk	26	24.5
	Intermediate-2 risk	39	36.8
	High risk	22	20.8
		Mean ± SD	
Ultrasonographic and laboratory findings	Spleen (mm)	162.25±33.59	
	Leukocyte (×10^3^/μL)	6.67±4.42	
	Hemoglobin (g/dL)	8.06±1.55	
	Neutrophil (×10^3^/μL)	4.87±3.95	
	Lymphocyte (×10^3^/μL)	1.57±1.02	
	Eosinophil (×10^3^/μL)	0.81±0.53	
	Basophil (×10^3^/μL)	0.81±0.87	
	Platelet (×10^3^ /μL)	191.60±167.90	

While a higher oxidative stress load was observed in patients with PM compared to the control group at the time of diagnosis, the oxidative stress load decreased with Ruxolitinib treatment (Table 2). 

**Table 2:** Evaluation of PM group's oxidative stress parameters in follow-up and comparison with the control group IMA: ischemia modified albumin, PM: primary myelofibrosis, SD: standard deviation *The difference between first month and at the time of diagnosis. **The difference between PM patients (at the time of diagnosis) and the control group. Statistically significant (p≤0.05) values were written in bold.

		PM (n=106)			Control (n=111)	
		At the time of diagnosis	First month after treatment	p-value*		p-value^**^
IMA	Mean ± SD	1.07±0.18	0.77±0.12	0.001	0.66±0.09	0.001
Native thiol	Mean ± SD	342.10±93.15	420.77±73.99	0.001	511.46±87.82	0.001
Total thiol	Mean ± SD	378.37±100.89	501.92±41.69	0.001	549.24±92.62	0.001
Disulfide	Mean ± SD	20.30±5.44	17.12±2.73	0.001	15.56±6.21	0.001
Disulfide/native thiol	Min-max (median)	1.7–13.4 (5.1)	2.1–6 (4.3)	0.001	0.7–6.5 (3.9)	0.001
Disulfide/total thiol	Min-max (median)	1.7–10.5 (4.7)	3–5 (4.1)	0.001	0.7–5.7 (3.6)	0.001
Native thiol/total thiol	Min-max (median)	78.9–108.5 (91.2)	85.2–92.1 (89.9)	0.043	71.3–98 (91.3)	0.229

Table 3 shows the changes in IMA and thiol parameters during follow-up according to genetic mutations. As seen in the table, Ruxolutinib reduces oxidative stress in all genetic categories.

**Table 3:** Evaluation of IMA and thiol parameters in follow-up according to genetic categories IMA: ischemia modified albumin, PM: primary myelofibrosis Statistically significant (p≤0.05) values were written in bold.

		Triple negative (n=6)	JAK2 (+) ASXL1 (−) (n=45)	MPL (+) ASXL1 (−) (n=10)	CALR (+) ASXL1 (−) (n=18)	ASXL1 (+) (n=27)
IMA	Before therapy	0.98±0.16	1.04±0.18	1.06±0.16	0.97±0.11	1.20±0.18
	First month after Ruxolitinib treatment	0.72±0.09	0.75±0.10	0.74±0.07	0.71±0.09	0.85±0.16
	P-value	0.028	0.001	0.005	0.001	0.001
Native thiol	Before therapy	386.35±43.27	334.72±99.85	372.18±79.08	339.07±61.35	335.43±110.61
	First month after Ruxolitinib treatment	471.30±52.66	411.24±76.62	436.53±52.40	403.94±81.07	430.81±72.44
	P-value	0.028	0.001	0.093	0.010	0.001
Total thiol	Before therapy	432.57±46.31	367.12±106.37	408.35±89.16	379.66±70.05	373.09±119.64
	First month after Ruxolitinib treatment	503.10±43.78	507.14±43.38	494.19±42.61	490.13±43.83	503.67±37.58
	P-value	0.028	0.001	0.059	0.001	0.001
Disulfide	Before therapy	23.12±5.94	18.88±4.88	22.90±4.25	21.97±5.2	19.95±6.20
	First month after Ruxolitinib treatment	15.47±3.80	16.99±2.50	16.38±3.25	16.64±2.64	18.28±2.46
	P-value	0.028	0.027	0.017	0.006	0.264
Native thiol/ total thiol	Before therapy	89.32±2.57	91.25±4.08	91.39±6.67	91.66±3.46	90.11±3.76
	First month after Ruxolitinib treatment	90.44±1.08	89.82±1.60	90.64±1.09	89.36±1.81	90.73±1.19
	P-value	0.249	0.011	0.799	0.064	0.442

According to DIPSS-plus risk categories, the decrease in IMA levels in the first month was statistically significant in all risk groups (p=0.001). It was found that native thiol and total thiol levels were lower in intermediate-2 and high-risk patients. In the first month after treatment, native thiol and total thiol measurements do not significantly differ. In paired comparisons, the change (increase) in the native thiol measurements of the high-risk group is higher than the low-risk and intermediate-1 risk groups (p=0.044, p=0.001, respectively) in the first month after treatment, compared to the time of diagnosis.

## Discussion

It is shown for the first time in this study that serum IMA and disulfide levels are significantly higher in patients with PM compared to healthy volunteers; native thiol and total thiol levels are substantially lower. Therefore, increased oxidative stress is indicated in PM. In other respects, it is demonstrated that serum IMA levels are significantly reduced, and native thiol and total thiol levels are significantly increased with Ruxolitinib treatment in patients with PM. Ruxolitinib diminishes the effects of oxidative stress. Moreover, in patients with the ASXL1 mutation and high-risk patients, the higher incidence of increased oxidative stress is also crucial. It may explain the worse prognosis in these patients.

Due to increased free oxygen radicals, oxidative stress causes DNA damage. Thus, it is thought that oxidative stress plays a role in the etiopathogenesis of many cancers [10]. It is set forth that, by further increasing clonal proliferation in the bone marrow in neoplasms such as myelofibrosis, increased oxidative stress may accelerate the transformation into acute leukemia [11]. We have detected high oxidative stress parameters in patients predicted to have a high probability of leukemic transformation based on mutation type, and prognostic risk scoring supports the critical role of oxidative stress in leukemic transformation. In vitro studies have proven that oxidative stress triggers hypoxia in bone marrow stem cells in patients with myelofibrosis [12]. Studies also show that increased hypoxia inducible factor-alpha (HIF-a) levels in these patients due to hypoxia can increase DNA damage by causing an unhealthy microenvironment in the bone marrow [13,14]. Increased IMA levels in these patients may indicate increased hypoxia in the bone marrow.

It has been reported in previous studies that, in myeloproliferative neoplasms, the presence of a Janus kinase 2 (JAK2) mutation is associated with an increase in free oxygen radicals and, consequently, DNA damage is increased [15]. In addition, this study has also shown that thiol compounds, which we consider as oxidative stress parameters, alter JAK activity [16]. In patients with myelofibrosis, Ruxolitinib, a JAK inhibitor, has also been shown to inhibit monocytic superoxide radical generation [17,18].

Ruxolitinib is a dual JAK1/2 specific inhibitor and has been approved by the FDA for the treatment of intermediate and high-risk myelofibrosis. Interestingly, in the Controlled Myelofibrosis Study with Oral JAK Inhibitor Treatment (COMFORT)-1 and COMFORT-2 studies, it was observed that the benefits of ruxolitinib were independent of the presence of the JAK2 mutation [19]. Again, this situation was attributed to the presence of mutations in myelofibrosis, ultimately affecting the JAK/STAT pathway. In addition, although it has been shown that the use of Ruxolitinib is effective in relieving symptoms and reducing spleen size, it has been shown that it does not significantly reduce the number of JAK2 alleles [20]. Ruxolitinib's oxidative stress-reducing effect demonstrated in this study can be attributed to reducing cytokine release rather than inhibiting the JAK/STAT pathway. The cytokine thesis is also supported by the fact that the effect of Ruxolitinib on constitutional symptoms is small.

It is known that the ASXL1 mutation, which is the most common epigenetic mutation in myelofibrosis, reduces overall survival not only in myelofibrosis but in all myeloid neoplasms [21]. The proliferation effect of ASXL1 is much greater than other mutations since it activates both the Akt/mTOR pathway and the JAK/STAT pathway [22,23]. Although the ASXL1 mutation's poor prognostic effect on myelofibrosis has been manifested, there are no studies about the impact of ASXL1 on oxidative stress in the literature. The conclusion we reached in this study is that the presence of the ASXL1 mutation causes more oxidative stress than the other genetic subtypes. The leukemic transformation that is more prominent in ASXL1 mutant cases may be associated with genomic instability and DNA damage due to increased oxidative stress. In the newly developed myelofibrosis risk scores, the ASXL1 mutation is considered a high-risk category [24]. With survival studies to be conducted in the future, integrating oxidative stress parameters into new scoring systems and thus more accurate prognosis may come into question.

Regarding the limitations of our study, the facts that we could not reevaluate the spleen size of the patients in the first month after treatment, the fact that we could not check for other epigenetic mutations (IDH1, TET2, etc.) except ASXL1, and the fact that we could not make comparisons based on prognostic scoring in which genetic features such as GIPSS and MIPSSv2 are prioritized, are the weaknesses of this study. Another limitation is the insufficient follow-up time for leukemic transformation. This study was conducted based on the knowledge that the rate of leukemic transformation is higher in patients with high-risk scores or ASXL1 positivity. Thus, we believe that new studies of prospective nature are needed.

## Conclusions

In patients with primary myelofibrosis, the oxidative stress load is markedly higher before Ruxolitinib treatment. This elevation is more prominent, especially in ASXL1-positive and high-risk patients. Ruxolitinib may be an effective treatment for reducing oxidative stress in patients with primary myelofibrosis.
